# Prevalence of Pulp Stones on Panoramic Radiographs in Patients with Chronic Systemic Diseases: A Cross-sectional Study

**DOI:** 10.5041/RMMJ.10557

**Published:** 2025-10-31

**Authors:** Shamimul Hasan, Tarun Sharma, Shazina Saeed, Mandeep Kaur, Virender Gombra, Rahnuma Masood

**Affiliations:** 1 Department of Oral Medicine and Radiology, Faculty of Dentistry, Jamia Millia Islamia, New Delhi, India; 2 Amity Institute of Public Health & Hospital Administration, Amity University, Noida, Uttar Pradesh, India

**Keywords:** Cardiovascular disorders, chronic periodontitis, diabetes mellitus, digital panoramic radiographs, pulp stones

## Abstract

**Introduction:**

Pulp stones (PS) are incidental, mostly asymptomatic, radiographic findings that may hinder endodontic therapy. They are observed as radiopaque aggregates within coronal or radicular pulp tissue on intraoral periapical, bite-wing, panoramic radiographs and cone-beam computed tomography images. This study aimed to evaluate PS prevalence in patients with cardiovascular disease (CVD), diabetes mellitus (DM), and chronic periodontitis (CP) as compared with controls, as a function of age and sex.

**Material and Methods:**

This cross-sectional study included 200 subjects: 50 healthy controls, and 50 patients each with CVD, DM, and CP. All participants underwent digital panoramic radiograph (orthopantomogram) (OPG) evaluation for the presence/absence, number, and location of PS.

**Results:**

Significant differences in PS prevalence were observed among the groups (*P*<0.01), with CVD patients showing the highest prevalence. Older individuals (>50 years) and first molars were most frequently affected. The maxillary arch showed a significantly higher prevalence than the mandible (odds ratio [OR]=1.45; 95% CI 1.22–1.72). The strongest risk factor was CVD (OR=7.38; 95% CI 5.20–10.47), followed by DM (OR=4.18; 95% CI 2.91–5.99) and CP (OR=4.16; 95% CI 2.88–6.00). Age was significantly associated with PS, while sex showed no association.

**Conclusion:**

The presence of PS, even among healthy controls, may serve as an adjunctive radiographic marker and could also alert dental practitioners to the possibility of underlying systemic disease.

## INTRODUCTION

Calcium deposits in the human body, referred to as calcification, are caused by the disorganized accumulation of calcium salts within soft tissue, leading to hardening. Such calcifications are found in the arteries, kidneys, lungs, and brain, as well as in dental pulp tissue.^1^ Pulp stones are distinct calcified aggregates within the coronal and radicular pulp of deciduous and permanent teeth, whether erupted or unerupted.^2^,^3^ They are usually incidental radiographic findings, typically asymptomatic unless found to compress nerves or blood vessels.^2^,^4^

Pulp stones are classified as true (dentin-like structure surrounded by odontoblasts) or false (calcified degenerating pulp tissue). They may also be described as free (completely surrounded by pulp), adherent (partially fused to the dentin), or embedded (completely enclosed by dentin).^2^,^5^,^6^

Pulp stones are most frequently found in molars, especially first molars, and less commonly in anterior teeth. They may complicate endodontic therapy by obstructing canal access leading to deflection, distortion, or fracture of endodontic instruments.^5^,^7^ The etiology of PS is multifactorial: aging, pulpal blood supply disruption, orthodontic movement, genetic predisposition, chronic irritation (deep caries, restorations, periodontal disease, trauma, occlusal forces), and possibly nanobacteria or bacterial toxins.^5^,^8^–^12^ Systemic conditions such as dentinogenesis imperfecta, dentin dysplasia, and Van der Woude syndrome have been linked to the development of PS.^5^,^13^ Importantly, PS formation may share mechanisms with vascular calcification in arteriosclerosis.^14^,^15^ Reported PS prevalence varies widely (8%–90%), depending on population, study design, sample size, and imaging modality.^3^,^7^,^9^–^11^,^16^,^17^

Various radiographic techniques, such as periapical radiographs,^16^,^18^,^19^ bite-wing radiographs,^10^,^11^,^14^,^16^ panoramic radiograph (orthopantomogram) (OPG),^1^,^6^,^11^,^20^,^21^ and cone-beam computed tomography (CBCT),^3^,^7^,^22^–^24^ have been employed to ascertain PS prevalence. However, radiographs often underestimate prevalence compared with histology.^3^,^5^,^6^,^11^,^18^,^25^

Several studies have suggested that PS may be associated with systemic diseases, especially cardiovascular disease (CVD), diabetes mellitus (DM), and chronic periodontitis (CP).^1^,^8^,^12^,^17^,^22^,^26^–^28^ These associations may indicate that PS are an early indicator of systemic disease. However, the literature remains inconsistent, and there are few comparative studies addressing these three conditions together.^17^,^22^,^27^–^29^

In light of this background, this study aimed to evaluate the prevalence of PS in different chronic systemic diseases. Specifically, we evaluated CVD, DM, and CP, and explored possible associations between PS and age, sex, tooth type, dental arch, side, and dental status, as well as whether PS are associated with these underlying systemic diseases.

## MATERIALS AND METHODS

This observational, cross-sectional study was conducted in the Outpatient Department of Oral Medicine and Radiology, Faculty of Dentistry, Jamia Millia Islamia, New Delhi. A total of 200 subjects (males and females), aged 20–70 years, were recruited. The participants were equally divided into four groups:

- Group I: 50 patients with a history of CVDs for >1 year.
- Group II: 50 patients with a history of DM for >1 year.
- Group III: 50 patients with clinical signs of periodontitis (plaque and calculus, bleeding on probing, pocket depth ≥4 mm, and tooth mobility).
- Group IV: 50 controls with no known history of CVDs, DM, or any other chronic systemic disease, no clinical signs of periodontitis. Patients with deep dental caries were excluded from participation as controls.

The study protocol received ethical approval from the Institutional Ethics Committee (IEC), Jamia Millia Islamia 19/6/447/JMI/IEC/2023. Voluntary written informed consent was obtained from all patients.

### Sample Size Estimation

The required sample size was calculated using the formula implemented by Nayak et al.:^17^


$$n=Z^{2}P(1-P)/d^{2}$$

where *n*=sample size; *Z*=*Z* statistic at 95% confidence interval (1.96); *P*=estimated prevalence of PS on radiographs (25% or 0.25); and *d*=precision of margin of error, expressed as a proportion (5%= 0.05).

The estimated sample size was approximately 188 patients. To ensure equal distribution, 200 subjects were included (50 per group).

### Inclusion and Exclusion Criteria

The inclusion and exclusion criteria for patients and controls are summarized in Table 1.

**Table 1:** Inclusion and Exclusion Criteria.

Group	Inclusion Criteria	Exclusion Criteria
Patients	Age 20–70 years (male/female) CVDs >1 year (hypertension, angina, myocardial infarction, heart surgery, congestive heart failure, cerebrovascular accident, hypercholesterolemia, arrhythmia*) DM >1 year Clinical signs of periodontitis: plaque and calculus, bleeding on probing, pocket depth ≥4 mm, tooth mobility	Unwilling to participate Age <20 or >70 years Both CVDs and DM present Pregnant/lactating Other systemic diseases (apart from CVDs/DM) Deep dental caries† Poor-quality radiographs‡
Controls	Age 20–70 (male/female) Good oral hygiene No clinical signs of periodontitis No known history of CVDs, DM, or any other chronic systemic disease No deep dental caries†	Same general exclusions as above

* Cardiac arrhythmias promote endothelial dysfunction, thrombogenesis, and systemic inflammation that accelerate atherosclerosis, hence they were part of the inclusion criterion for this study.

† Deep dental caries was excluded as this can act as a chronic, low-grade irritant to the pulp tissue, triggering inflammation and reparative responses that may lead to pulp stone formation.

‡ Poor quality radiographs were defined as images with inadequate clarity or contrast, where radiopaque

We further narrowed the sample by only including patients with pain in both maxillary and mandibular posterior teeth, and with generalized periodontitis.

### Study Procedure

Demographic details and medical history were recorded on a structured proforma (Supplement). A detailed clinical evaluation was performed under adequate lighting, with the patient seated comfortably in a dental chair in a supine position, following infection control protocols.

Eligible patients then underwent digital panoramic radiography (orthopantomogram, OPG) using the Carestream (Kodak) CS 1800 dental X-ray machine (Carestream Health, Rochester, NY, USA), with exposure parameters of 68–74 kVp and 8–10 mA for 15.8–16 seconds. Radiographs were obtained in accordance with radiation protection protocols. These radiographs were part of the patient’s diagnostic work-up, and no additional ones were obtained for the purposes of the study. The radiographs were analyzed and interpreted for the presence/absence, number, and location of PS, including distribution by jaw (maxilla/mandible), tooth type, and side (right/left).

### Data Analysis

The data obtained were entered into Microsoft Excel and analyzed using SPSS version 20.0 (IBM Corp. Armonk, NY, USA). Two-tailed *t*-tests were performed, and a *P* value of <0.05 was considered statistically significant. Chi-square tests were conducted for comparisons between patient groups.

## RESULTS

This study examined PS prevalence among 200 subjects of both sexes, distributed across four groups: individuals diagnosed with CVDs, DM, CP, and a healthy control group. Each group comprised 50 participants aged 20–70 years. The descriptive characteristics of the participants and teeth examined are summarized in Table 2.

**Table 2:** Descriptive Characteristics of Study Participants by Group (Age, Sex, Jaw, and Tooth Type Distributions).

Variable	Age (Years)			Sex		Number of Teeth Examined								
	20–35	36–50	>50	Male	Female	Jaw		Tooth Type						Total
						Mx	Mn	Inc	C	1st PM	2nd PM	1st M	2nd M	
CVD (n=50), *n* (%)	3 (6.0)	17 (34.0)	30 (60.0)	27 (54.0)	23 (46.0)	519 (45.3)	627 (54.7)	329 (28.7)	154 (13.4)	158 (13.8)	145 (12.7)	182 (15.9)	178 (15.5)	1146 (100.0)
DM (n=50), *n* (%)	3 (6.0)	8 (16.0)	39 (78.0)	29 (58.0)	21 (42.0)	602 (48.8)	632 (51.2)	205 (16.6)	211 (17.1)	180 (14.6)	202 (16.4)	221 (17.9)	215 (17.4)	1234 (100.0)
CP (n=50), *n* (%)	6 (12.0)	10 (20.0)	34 (68.0)	26 (52.0)	24 (48.0)	509 (48.6)	538 (51.4)	174 (16.6)	162 (15.5)	172 (16.4)	172 (16.4)	178 (17.0)	189 (18.1)	1047 (100.0)
Control (n=50), *n* (%)	6 (12.0)	13 (26.0)	31 (62.0)	26 (52.0)	24 (48.0)	550 (48.8)	576 (51.2)	178 (15.8)	187 (16.6)	203 (18.0)	189 (16.8)	191 (17.0)	178 (15.8)	1126 (100.0)
Total (n=200), *n* (%)	18 (9.0)	48 (24.0)	134 (67.0)	108 (54.0)	92 (46.0)	2180 (47.9)	2373 (52.1)	886 (19.5)	714 (15.7)	713 (15.7)	708 (15.6)	772 (17.0)	760 (16.7)	4553 (100.0)

C, canines; CP, chronic periodontitis, CVD, cardiovascular disease; DM, diabetes mellitus; Inc, incisor; M, molar; Mn, mandible; Mx, maxilla; *n*, number; PM, premolar.

Note: Columns 2–6: percentages calculated within each disease group; Columns 7–14: percentages calculated for the number of teeth examined within each category.

The overall prevalence of PS was evaluated both per patient and per tooth. As shown in Table 3, significant differences (*P*<0.01) were observed among the study groups, with the highest prevalence in CVD patients, followed by DM and CP, and the lowest in the controls. Participants over 50 years have a markedly higher PS prevalence compared to younger and middle-aged individuals (*P*<0.01), with the CVD group showing the greatest prevalence in this category. No significant association was found between sex (*P*=0.214) or side (right versus left; data not included in the tables) and PS prevalence.

**Table 3:** Prevalence of Pulp Stones in Study Groups by Age and Sex.

Variable	Number of Patients	Prevalence of Pulp Stones, *n* (%)	Patients with Pulp Stones by Age and Sex, *n* (%)				
			Age Group			Sex	
			20–35 y	36–50 y	>50 y	Male	Female
CVD	50	17 (34.0)	4 (23.5)	5 (29.4)	8 (47.0)	9 (52.9)	8 (47.1)
DM	50	11 (22.0)	3 (27.2)	3 (27.2)	5 (45.5)	5 (45.5)	6 (54.5)
CP	50	10 (20.0)	1 (10.0)	3 (30.0)	6 (60.0)	6 (60.0)	4 (40.0)
Control	50	4 (8.0)	1 (25.0)	1 (25.0)	2 (50.0)	1 (25.0)	3 (75.0)
TOTAL	200	42 (21.0)	9 (21.4)	12 (28.6)	21 (50.0)	21 (50.0)	21 (50.0)
*P* value*		0.016	0.97			0.67	

* *P* values from chi-square tests comparing CVD, DM, CP, and Control within each variable.

CP, chronic periodontitis; CVD, cardiovascular diseases; DM, diabetes mellitus; *n*, number; y, year.

At the tooth level (Table 4), PS prevalence also varied significantly across groups (*P*<0.001). By jaw, the distribution did not differ significantly (*P*= 0.536). According to tooth type, molars were most affected, particularly the first molar, which displayed the highest prevalence (3.03%, 138 affected teeth).

**Table 4:** Prevalence of Pulp Stones in Study Groups by Jaw and Tooth Type.

Variable	Teeth Examined, *n*	PS Prevalence, *n* (%)	Teeth with PS by Jaw, *n* (%)		Teeth with PS by Tooth Type *n* (%)					
			Mx	Mn	Inc	C	1st PM	2nd PM	1st M	2nd M
CVD	1146	240 (20.9)	137 (57.1)	103 (42.9)	23 (9.6)	29 (12.1)	31 (12.9)	43 (17.9)	58 (24.2)	56 (23.3)
DM	1234	161 (13.0)	102 (63.4)	59 (36.6)	22 (13.7)	25 (15.5)	21 (13.0)	22 (13.7)	33 (20.5)	38 (23.6)
CP	1047	136 (13.0)	78 (57.4)	58 (42.6)	11 (8.0)	16 (11.8)	26 (19.1)	21 (15.4)	34 (25.0)	28 (20.5)
Control	1126	39 (3.4)	21 (53.8)	18 (46.1)	4 (10.3)	5 (12.8)	4 (10.2)	6 (15.4)	13 (33.3)	7 (17.9)
TOTAL	4553	576 (12.6)	338 (58.7)	238 (41.3)	60 (10.4)	75 (13.0)	82 (14.2)	92 (16.0)	138 (24.0)	129 (22.4)
*P* value			0.54		0.75					

* *P* values from chi-square tests comparing CVD, DM, CP, and Control within each variable.

%, percent of teeth with pulp stones in that group; C, canines; CP, chronic periodontitis; CVD, cardiovascular disease; DM, diabetes mellitus; Inc, incisor; M, molar; Mn, mandible; Mx, maxilla; *n*, number; PM, premolar.

Region-wise analysis revealed notable disparities (*P*<0.001) in PS prevalence across the maxillary arch, with the CVD group exhibiting the highest prevalence (26.4%). Tooth-specific prevalence varied significantly among the study groups (*P*<0.05) (Table 5). The CVD patients showcased the highest prevalence, followed by DM and CP groups. However, the marked statistical significance was largely due to the very low prevalence observed in the control group, particularly in individuals younger than 50 years. Among controls PS were mainly identified in those over 50 years of age.

**Table 5:** Comparative Evaluation of the Prevalence of Pulp Stones by Region and Tooth.

Variable	Cardiovascular Diseases		Diabetes Mellitus		Chronic Periodontitis		Control		*P* Value

	Teeth Examined (*n*)	Pulp Stones(*n*, %)	Teeth Examined (*n*)	Pulp Stones(*n*, %)	Teeth Examined (*n*)	Pulp Stones(*n*, %)	Teeth Examined (*n*)	Pulp Stones(*n*, %)	

**Jaw**									
Mx	519	137 (26.4)	602	102 (16.9)	509	78 (15.3)	550	21 (3.8)	<0.001

Mn	627	103 (16.4)	632	59 (9.3)	538	58 (10.7)	576	18 (3.1)	<0.001

**Tooth Type**									
Inc	329	23 (6.90)	205	22 (10.7)	174	11 (6.3)	178	4 (2.2)	0.012

C	154	29 (18.8)	211	25 (11.8)	162	16 (9.8)	187	5 (2.6)	<0.001

1st PM	158	31 (19.6)	180	21 (11.6)	172	26 (15.1)	203	4 (1.9)	<0.001

2nd PM	145	43 (29.6)	202	22 (10.8)	172	21 (12.2)	189	6 (3.1)	<0.001

1st M	182	58 (31.8)	221	33 (14.9)	178	34 (19.1)	191	13 (6.8)	<0.001

2nd M	178	56 (31.4)	215	38 (17.6)	189	28 (14.8)	178	7 (3.9)	<0.001

C, canines; Inc, incisor; M, molar; Mn, mandible; Mx, maxilla; *n*, number; PM, premolar.

Further examination of independent variables (study group, age, sex, jaw position, region, and tooth type) identified CVD as a significant risk factor for PS development, followed by DM and CP (Table 6). Increasing age was significantly associated with a higher risk of PS, particularly in participants over 50 years. The maxilla displayed a higher risk for PS compared to the mandible, and molars (especially the first and second molars) presented the highest risk among tooth types, while canines and first premolars did not differ significantly from incisors. No significant associations were observed between gender or side (right versus left) and PS prevalence.

**Table 6:** Univariate Analysis of Risk Factors for Pulp Stone Development (Odds Ratios).

Variable	Odds Ratio	95% CI	*P* Value
**Study Group**			
Control	**Reference**		
	
CVD	7.38	5.20–10.47	<0.001
	
DM	4.18	2.91–5.99	<0.001
	
CP	4.16	2.88–6.00	<0.001

**Age (years)**			
20–35	**Reference**		

36–50	2.65	1.95–3.58	<0.001

>50	6.62	5.01–8.74	<0.001

**Sex**			
Female	**Reference**		

Male	1.06	0.89–1.25	0.491

**Jaw**			
Mn	**Reference**		

Mx	1.45	1.22–1.72	<0.001

**Tooth Type**			
Inc	**Reference**		
	
C	1.25	0.89–1.76	0.190
	
1st PM	1.37	0.98–1.92	0.060
	
2nd PM	1.54	1.11–2.14	0.009

1st M	2.34	1.72–3.17	<0.001

2nd M	2.18	1.60–2.97	<0.001

C, canines; CP, chronic periodontitis; CVD, cardiovascular diseases; DM, diabetes mellitus; Inc, incisor; M, molar; Mn, mandible; Mx, maxilla; *n*, number; PM, premolar.

## DISCUSSION

### Background and Definitions

Pulp calcifications were first described as dental pulp nodules in 1921 and later termed “denticles.”^13^ Currently, they are broadly classified into two types: pulp stones (PS) and pulp canal obliteration (PCO). Pulp stones are localized mineral deposits within the pulp, found in both carious and non-carious, deciduous, permanent, and even unerupted teeth, most commonly in the coronal pulp, but also seen in the radicular pulp. They are typically asymptomatic and identified incidentally on radiographs.

Pulp canal obliteration involves radiographic narrowing or complete loss of the root canal space and is often associated with yellowish tooth discoloration. It commonly occurs in teeth with a history of trauma and, like pulp stones, is usually asymptomatic and discovered during routine clinical and radiographic evaluation.^30^

### Diagnostic Methods

The present study used digital OPGs to detect PS in chronic systemic diseases (CVDs, DM), CP, and controls. Digital OPGs have become an indispensable component of dental investigations in various healthcare or clinical settings. Both the jaws and the supporting structures may be quickly visualized using minimal ionizing radiation.^7^,^9^ They further facilitate easy electronic storage and transfer of these images.^25^ While several studies have used OPGs to evaluate PS prevalence,^1^,^5^,^7^,^9^,^12^,^24^,^25^ other radiographic techniques have also been employed, including periapical radiographs,^16^,^18^,^19^ bite-wing radiographs,^9^,^10^,^13^,^15^ and CBCT.^3^,^7^,^23^,^24^

Radiographs are regarded as a non-invasive tool for the assessment of PS, although very small calcifications (<200 μm) may not be detected.^3^,^5^,^6^,^10^,^17^,^18^,^25^,^31^ Histologic assessment has demonstrated a higher prevalence of PS than radiographic examination.^5^,^6^,^8^–^11^,^14^,^15^

### Clinical Significance of Pulp Stones

Pulp stones may pose problems in endodontic therapy, as they hinder the root canal course, causing technical difficulties in locating, accessing, and preparing root canals. In addition, the PS may also cause file tip distortion and may impede the disinfection/irrigation process.^1^–^5^,^9^,^11^,^31^

Zhang et al. recently pointed out that although pulp stones are typically asymptomatic they may occasionally be associated with trigeminal neuralgia, even in teeth with vital pulp and no evident pathology.^32^ This suggests that mechanical compression of intrapulpal nerve fibers by calcifications could play a role in neuropathic pain. However, as the pulp remains vital, specific dental intervention is typically not required.

In forensic odontology, radiographic matching of PS patterns, along with other recorded characteristics, may render pivotal details in the identification of deceased individuals.^9^–^11^

### Associations with Systemic Disease

While pulp calcification is typically considered a localized occurrence, recent research has hinted at a potential association between pulp calcification and systemic disorders. Pulp canal calcification could potentially indicate systemic calcification, a prevalent characteristic found in various systemic diseases.^33^

Pulp stones have been associated with a number of system conditions, including CVDs,^1^,^8^,^12^,^16^,^17^,^20^,^21^,^34^–^36^ DM,^8^,^17^,^29^ renal disorders,^1^,^8^,^12^,^26^ autoimmune disorders,^17^ and CP.^27^,^28^ Some authors have suggested that PS may serve as an early diagnostic marker of systemic diseases.^7^,^8^,^20^,^22^,^25^ However, the literature remains inconsistent, and few comparative studies have addressed these three conditions together.^17^,^22^,^27^–^29^

These systemic ailments amend the congruous equilibrium among the oral microbial flora and result in oral dysbiosis (sudden upsurge in the virulent bacterial strains), thereby facilitating an environment favorable for inflammatory events (raised pro-inflammatory cytokines levels). Oral dysbiosis not only increases the risk of periodontal diseases but also exacerbates underlying systemic conditions.^22^,^25^,^27^,^28^,^37^,^38^

Therefore, radiographs of the jaws and teeth are clinically important for identifying PS that may point to an underlying systemic condition. Nevertheless, confirmation requires a thorough clinical assessment and comprehensive medical history.^39^

### Pathogenesis

The calcification observed in different areas such as the kidneys, joints, teeth, and atherosclerotic plaque is believed to be primarily composed of calcium phosphate crystals, which trigger an acute immune reaction, resulting in inflammation within the arteries. These occurrences are a major contributing factor to ischemic heart disease, resulting in substantial mortality and morbidity. Another theory suggests that calcifying nanoparticles, specifically nanobacteria, play a crucial role in the pathological calcifications observed in gallstones, joint calcification, renal calculi, atherosclerotic plaque, and PS.^22^

Calcifying nanoparticles (CNPs), also known as nanobacteria, contribute to pulp stone formation primarily by disrupting dental pulp cells and initiating calcification pathways. They interact with calcium and phosphate ions present in the pulp tissue to form inorganic precipitates, which act as nucleation sites for bio-mineralization. These sites promote the deposition and growth of calcium phosphate crystals, ultimately leading to the formation of pulp stones.^40^,^41^

Recent literature suggests that osteopontin, a newly identified component of atherosclerotic plaques, plays a critical role in plaque calcification. Moreover, there is evidence that osteopontin, produced by macrophages, has a critical role in forming calcification centers within the carotid and renal arteries.^21^

The role of osteopontin in pulp stone formation appears to be significant, primarily related to its localization and properties. Osteopontin, a glycophosphoprotein produced by dental pulp cells, is mainly found at the calcification front of pulp stones, particularly on the outer surface of pulp stones. It plays a key role in regulating crystal growth and nucleation, thereby facilitating calcification of the organic matrix. Furthermore, its localization at the calcification front indicates that osteopontin could act as a mediator in the formation and progression of PS, possibly by interacting with mineralized tissue components and influencing crystal size and organization.^42^,^43^

### Interpretation of Study Findings

#### Cardiovascular Disease (CVD)

Cardiovascular diseases continue to be the leading causes of global mortality and significantly contribute to diminished health and increased healthcare expenditures.^44^ Pulp stones are regarded as an initial risk factor identified during the onset of CVDs.^21^

Our study demonstrated an overall prevalence of 34% for PS in the CVD group. Comparable prevalence rates of PS were also found in other studies.^12^,^22^,^45^ Conversely, some studies showed a lower prevalence of PS,^17^,^20^ while others reported higher prevalence rates.^1^,^21^,^35^,^46^ Our data indicated that CVDs pose a significant risk for PS formation compared to healthy subjects.

The tooth-specific prevalence of PS in the CVD group was 20.94%. The maxillary arch (11.9%) and the 1st molar tooth (5.06%) were associated with a higher prevalence. Our study findings are consistent with the published literature.^12^,^17^,^22^

#### Diabetes Mellitus

Hyperglycemia can trigger the production of reactive oxygen species and compromise antioxidant defenses, leading to a range of complications in DM. Diabetic patients display changes in the dental pulp structure, decreased pulp capacity, and diminished vascularity. Pronounced inflammatory cell infiltration and inflammatory mediator expression have also been observed.^47^

The present study demonstrated an overall PS prevalence of 22% in the DM group. Comparable prevalence rates of PS were also noted in other studies.^22^,^29^ The tooth-specific prevalence of PS in the diabetic group was 13.04%. Interestingly, this contrasts with the findings of previous studies,^17^,^22^ which reported lower tooth-specific prevalence of PS. Nevertheless, our study indicated that DM is an important risk factor for PS formation. One possible reason for this finding could be that diabetic patients experience vascular changes such as obliterative endarteritis and thickening of vessel walls (diabetic microangiopathy). This reduces pulpal blood flow, leading to ischemia and fibrosis. While these changes resemble age-related degeneration, in DM they occur earlier and independently due to chronic hyperglycemia-induced vascular damage. This compromised blood supply predisposes the pulp to dystrophic calcification and increases the likelihood of pulp stone formation. In addition, higher blood glucose levels may activate osteopontin, further promoting pathological mineralization.^22^,^29^,^48^

#### Chronic Periodontitis

Pulp calcifications have also been associated with chronic tissue inflammation, such as periodontal disease, indicating a potential role of inflammation in PS formation.^27^ Periodontal diseases may disrupt pulp vascularity and nutrition, resulting in a reduction in cellular elements and increased calcification.^49^ The present study documented a 20% prevalence of PS among individuals with chronic periodontitis, which corresponds to the findings of Kantaputra et al.^13^ However, a higher prevalence of PS in periodontitis patients was observed by others.^27^,^28^,^49^

#### Age

Considering the independent variables, our study reported that most of the PS were seen in patients >50 years of age, irrespective of the study group. With advancing age, there is a natural tendency for structural changes in the dental pulp, including decreased cellularity, reduced vascular supply, increased fibrosis, and chamber narrowing. These age-related degenerative changes create a favorable environment for dystrophic calcification.^50^ The exact mechanism may involve altered pulpal metabolism, reduced clearance of metabolic by-products, and chronic low-grade inflammation, all of which can promote calcific nodule formation.^51^

This variation in the reported age distribution of pulp stones may be attributed to differences in study design (hospital-based versus community-based sampling), population characteristics (including ethnic diversity, systemic conditions, and oral health practices),^10^–^12^ as well as diagnostic criteria (whether diffuse or discrete calcifications are considered). The radiographic technique employed also plays a role, since smaller calcifications often go undetected on 2D imaging but can be readily identified with CBCT.^7^

A higher occurrence of PS in older subjects has also been reported in other studies.^10^,^11^,^17^,^22^,^25^,^37^,^38^,^33^ However, our findings are in contrast to other studies, where a higher prevalence of PS was seen in younger subjects.^8^,^9^,^20^

#### Sex

Our study revealed a comparable likelihood of PS formation in both sexes, consistent with observations from other studies.^6^,^7^ However, some studies have noted PS to be more prevalent in females than in males,^9^,^11^,^12^,^18^,^19^,^25^ while others have observed a higher prevalence among males.^23^,^46^ These findings remain inconclusive and merit further investigation.

#### Jaw and Tooth Type

Our study demonstrated a higher prevalence of PS in the maxilla and in molars across all groups, suggesting that these sites are at greater risk for PS formation. These data are consistent with several previous studies.^6^,^9^,^11^,^12^,^17^,^19^,^22^,^25^,^46^ However, one study has reported a higher prevalence in the mandible.^8^

The higher occurrence of calcium deposits in the molars may be related to the rich vascularity of their pulp tissue. Moreover, the early eruption of the first molars, together with subsequent masticatory forces and occlusal stress, may contribute to increased pulp calcification.^16^

Although the maxillary arch has a richer vascular supply, its higher PS prevalence may also reflect anatomical and functional factors such as larger pulp volumes and a longer period of exposure, beginning with the early eruption of molars and extending through cumulative irritants and occlusal forces, rather than reduced perfusion alone.

The lower occurrence of PS in anterior teeth, despite their rich vascular supply, can be attributed to their simpler root canal anatomy, smaller pulp chambers, and reduced exposure to chronic irritants such as deep caries or restorations. In contrast, posterior teeth, particularly molars, possess larger and more complex canal systems and are more frequently subjected to irritants, making them more susceptible to pulp inflammation and subsequent calcification.

### Limitations and Future Directions

Our study was carried out at a single center, potentially limiting the generalization of the results to the broader population. Therefore, conducting multicenter studies with larger sample sizes is essential to achieve more precise and representative results.

The spatial resolution and 2-dimensional nature of OPG could lead to the misinterpretation of pulp stones, especially in their early calcification stages (<200 μm), thus overestimating or underestimating the true frequency of PS.

Another limitation is that there is no mention of a size cutoff or standard measurement for confirming pulp stones or differentiating between solitary versus conglomerate stones. Hence, it is imperative to use three-dimensional radiographic techniques like CBCT, as they offer detailed visualization with individual assessment of each tooth.

The cross-sectional design of our study also poses a limitation; therefore, longitudinal studies with regular follow-ups are necessary.

## CONCLUSION

Pulp stones may be an incidental finding on routine panoramic radiographs. The radiographic presence of PS should be able to help the dental surgeon in identifying patients with hitherto undiagnosed systemic diseases. Panoramic radiographs may be employed as a rapid, economical, mass screening investigation tool for identification of systemic disorders on a large-scale basis, perhaps many years before the disease’s symptoms are apparent.

Our findings suggest that radiographic detection of PS is associated with CVDs, DM, and CP, and may provide useful data for identifying individuals at higher risk of these conditions. As such, the importance of dental radiographic assessment in early detection and preventive strategies for underlying systemic conditions should not be underestimated.

The study also underscores the significance of early detection and treatment of both dental and underlying systemic conditions, showcasing the potential impact dentists can have on the overall health and well-being of patients.

## Supplementary Information



## Abbreviations

CBCT: cone-beam computed tomography
CP: chronic periodontitis
CVD(s): cardiovascular disease(s)
DM: diabetes mellitus
(OR): odds ratio
OPG: panoramic radiograph (orthopantomogram)
PS: pulp stones.
