# Supplementary material for: Prevalence of Pulp Stones on Panoramic Radiographs in Patients with Chronic Systemic Diseases: A Cross-sectional Study
Source: Rambam Maimonides Med J. 2025 Oct 31;16(4):e0022. doi: 10.5041/RMMJ.10557 (PMC12591515; doi:10.5041/RMMJ.10557)
Supplement: Supplementary file 1 [file rmmj-16-4-e0022_Supplement.pdf]

SUPPLEMENTARY MATERIAL

This appendix has been provided by the authors for the benefit of readers

Supplement to Prevalence of Pulp Stones on Panoramic Radiographs in Patients with Chronic Systemic Diseases: A Cross-Sectional Study

Hasan S, Sharma T, Saeed S, Kaur M, Gombra V, Masood R. Prevalence of Pulp Stones on Panoramic Radiographs in Patients with Chronic Systemic Diseases: A Cross-Sectional Study. Rambam Maimonides Med J 2025;16 (4):e0022. doi:10.5041/RMMJ.10557

STUDY QUESTIONNAIRE

Patient Information

OPD No.: ________

Name: _____________________________

Age: ________

Sex: Male / Female

Address: ___________________________

Phone Number: _____________________

I. Cardiovascular Disorder History

Coronary artery disease	☐Yes	☐No
Hypertension	☐Yes	☐No
Arrhythmia	☐Yes	☐No
Heart failure	☐Yes	☐No
Valvular condition	☐Yes	☐No

Other: ____________________________

If Yes, duration (years):

☐1–5 ☐6–10 ☐11–20 ☐21–30 ☐>30

II. Diabetes Mellitus History

☐ Yes ☐ No

If Yes, duration (years):

☐ 1–5 ☐ 6–10 ☐ 11–15 ☐ 16–20 ☐ 21–30

III. Other Systemic Diseases

____

IV. Family History of Systemic Disorders

Clinical Examination Findings

1. Oral Hygiene Status (plaque and calculus):

☐ Mild ☐ Moderate ☐ Severe

2. Probing Depth (mm):

Tooth No. __________ | Depth: ______ mm

3. Tooth Mobility:

Tooth No. __________ | Grade: __________

Radiographic Examination Findings

Pulp stones: ☐ Present ☐ Absent

Location of pulp stones (relation to teeth):

- Maxillary teeth ☐
- Mandibular teeth ☐

Pulp stones detected in tooth type:

☐ Incisor ☐ Canine ☐ 1st premolar ☐ 2nd premolar ☐ 1st molar ☐ 2nd molar

Location of pulp stone (side): ☐ Right ☐ Left
